# A Diverse Community of Metal(loid) Oxide Respiring Bacteria Is Associated with Tube Worms in the Vicinity of the Juan de Fuca Ridge Black Smoker Field

**DOI:** 10.1371/journal.pone.0149812

**Published:** 2016-02-25

**Authors:** Chris Maltman, Graham Walter, Vladimir Yurkov

**Affiliations:** Department of Microbiology, University of Manitoba, Winnipeg, Canada; Universite Pierre et Marie Curie, FRANCE

## Abstract

Epibiotic bacteria associated with tube worms living in the vicinity of deep sea hydrothermal vents of the Juan de Fuca Ridge in the Pacific Ocean were investigated for the ability to respire anaerobically on tellurite, tellurate, selenite, selenate, metavanadate and orthovanadate as terminal electron acceptors. Out of 107 isolates tested, 106 were capable of respiration on one or more of these oxides, indicating that metal(loid) oxide based respiration is not only much more prevalent in nature than is generally believed, but also is an important mode of energy generation in the habitat. Partial 16S rRNA gene sequencing revealed the bacterial community to be rich and highly diverse, containing many potentially new species. Furthermore, it appears that the worms not only possess a close symbiotic relationship with chemolithotrophic sulfide-oxidizing bacteria, but also with the metal(loid) oxide transformers. Possibly they protect the worms through reduction of the toxic compounds that would otherwise be harmful to the host.

## Introduction

Bacterial respiration on oxyanions of metal(loid)s is known [1], however, it was not believed to be widespread. Due to the high toxicity, especially of tellurium oxides, it has long been believed they have no significant (if any, in the case of Te) role in biological processes. However, microbes have adapted and evolved to incorporate oxyanions of Te, Se, and V into metabolic processes, especially in metal(loid) rich environments [2]. In regards to dissimilatory electron transport to metal(loid)s, strong support comes from the physical/chemical features of the redox couples for Te, Se, and V oxides (TeO_3_^2-^/Te = 0.827 V; TeO_4_^2-^/TeO_3_^2-^ = 0.885 V; SeO_3_^2-^/Se = 0.885 V; VO_2_^+^/VO^2+^ = 1.000 V). Although highly toxic, they are more favorable for anaerobic respiration than that of SO_4_^2-^/HS^-^ (-0.217 V) couple widely used by sulfate reducers [3]. No dissimilatory anaerobic reduction of Te oxyanions was known until 2006, when strain ER-Te-48 from a deep sea hydrothermal vent tube worm was found to be capable of anaerobic tellurate based respiration [4]. Since then, four other bacteria have been shown to respire on Te oxides [5–7]. The dissimilatory use of Se and V oxides has been known for some time, however, it is limited to only a select few species [4–19]. The majority are halophiles from locales lacking any detectable metal(loid)s, suggesting the ability to respire on oxides was not directly evolved for survival.

Deep sea hydrothermal vents, so-called Black Smokers, are geological formations, which release subterranean seawater that has been superheated to more than 400°C by magma pockets beneath the sea floor. Through this process, metal(loid)s are mobilized from the crustal basalts, highly enriching the vent plumes [20, 21]. The harsh environment suggests life there should be scarce, however, numerous unique organisms call this ecological niche home. The sulfide and tube worms surrounding vents are of particular interest with regards to bacterial-metal(loid) interactions. Due to the proximity to the plume waters, they and their associated symbiotic microbes are in close contact with elevated levels of metal(loid)s [22]. These animals harbor a community of metal resistant bacteria [23], indicating that the microbial population does experience, and has adapted to metal(loid) exposure. Such conditions offer the perfect environment for the evolution of biological processes dependant on metal(loid)s. Another feature of these worms providing selective pressure in favor of bacteria capable of metal(loid) resistance/respiration is their vanadium enriched blood [24]. Since these creatures possess conditions ideal for dissimilatory metal(loid) reduction, it is not surprising their epibionts gave us not only the first example of anaerobic respiration on Te oxides [4], but also on metavanadate [17], and orthovanadate [4].

As mentioned prior, there are very few known examples of microbes utilizing Te, Se, or V oxides as terminal electron acceptors during anaerobic growth. They are spread out among different genera [4–19], suggesting metal(loid) oxide respiring microbes are phylogenetically diverse and not limited to a single taxonomic group. In this study, we investigated 107 epibiotic isolates from the vent tube worms *Paralvinella sulfincola* and *Ridgea piscesae* of the Axial Volcano (AV) caldera and Explorer Ridge (ER) vent field of the Juan de Fuca Ridge [4] for the ability to respire anaerobically on tellurite, tellurate, selenite, selenate, metavanadate, and orthovanadate. Partial sequencing of the 16S rRNA gene was then carried out to determine their phylogenetic diversity.

## Materials and Methods

### Growth and respiration with metal(loid) oxides

Sampling and collection of sulfide tube worms (*R*. *piscesae*) and tube worms (*P*. *sulfincola*) from Axial Volcano (Hell Vent: 45°56’00”N, 130°00’51”W; 1,543 m) and Explorer Ridge (Lucky Find: 49°45’38”N, 130°15’23”W; 1,791 m) of the Juan de Fuca Ridge in the Pacific Ocean in 2003 was as previously published [4]. Tissue from the worms was rinsed, homogenized, and used for inoculation of enrichment cultures. 107 metal(loid) reducing epibiotic bacterial strains were isolated as described [4]. Each was grown aerobically at 28°C in the dark on rich organic (RO) [25] plates containing 2% NaCl and used to inoculate Balch tubes of anaerobic metal(loid) respiration (AMR) liquid medium, containing (g/l): KH_2_PO_4_, 0.5; NH_4_Cl, 0.5; CaCl_2_, 0.1; yeast extract, 1.0; lactate, 1.0; and MgSO_4_, traces. Vitamin and trace microelements solutions [25] were added at 2 ml/l. Medium was amended with one of tellurite, tellurate, selenite, selenate (100 μg/ml), or metavanadate, orthovanadate (500 μg/ml) with a headspace of N_2_. Tubes were incubated at 28°C in the dark and monitored for respiration over two weeks. A representative strain was chosen for each oxide reducing group. Aerobically grown cells of ER-Te-40B, ER-Te-57, AV-Te-18, ER-V-8, AV-V-4, and ER-Te-41 were used to inoculate 120 ml crimp-sealed bottles containing 100 ml of AMR medium with one of tellurite, tellurate, selenite, selenate, metavanadate or orthovanadate at the concentrations listed above, under a headspace of N_2_. Control tubes were not supplemented with any of these oxides. Protein yield was measured by Bradford assay [26]. ATP was measured using an ATP Bioluminescence Kit from Sigma-Aldrich. All experiments were performed in triplicate.

### Phylogenetic analysis

Genomic DNA was extracted from pure cultures of each isolate as published [27]. Partial 16S rRNA gene amplification by PCR was carried out using universal bacterial primers [28], in 50 μl reaction volumes containing: 25 μl DreamTaq PCR Master Mix, 0.25 μM of each primer, and between 10 and 50 ng of DNA. The amplification cycle was as follows: Initial denaturing at 95°C for 5 min, denaturing at 95°C for 30 sec, annealing at 46°C for 30 sec, extension at 72°C for 1.5 min for 35 cycles with a final extension at 72°C for 10 min, ending with a hold at 7°C. Preparation of the PCR products was as described [29]. Samples were sequenced by the Manitoba Institute of Cell Biology. The nucleotide sequences were edited and phylogenetic relatedness determined as reported [29]. All sequences were deposited in Genbank under the accession numbers provided in S1 Table. Maximum likelihood phylogenetic trees were created using Phylogeny.fr [30].

## Results and Discussion

### Growth and reduction with metal(loid) oxides

**Fig 1:**
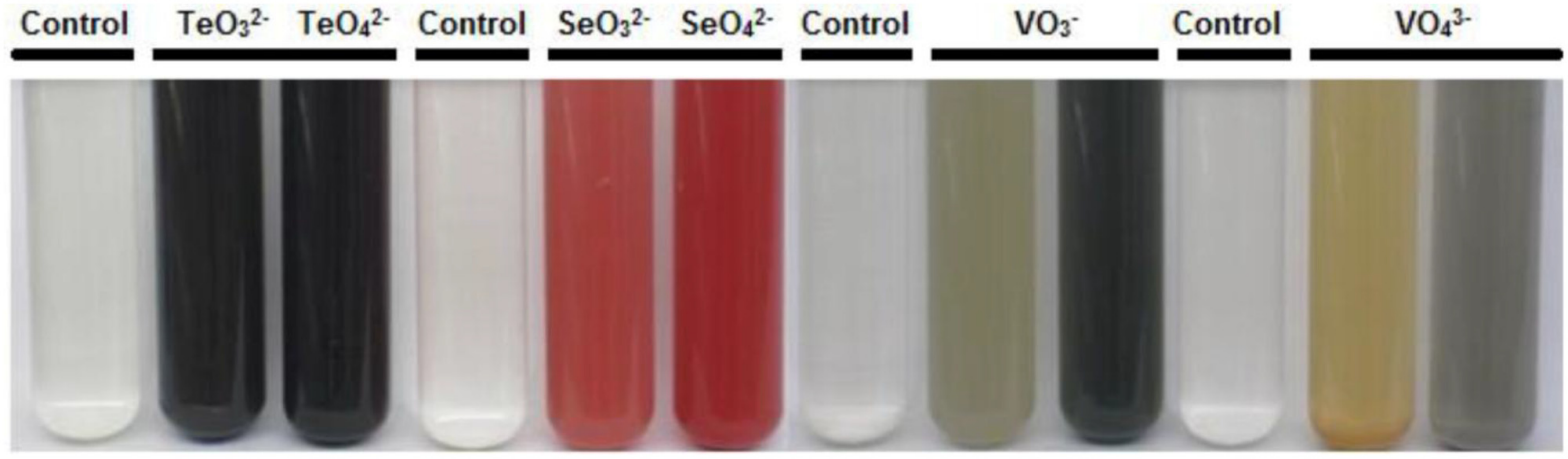
Anaerobic respiration resulting in visible reduction of tellurite, (strain ER-Te-40B), tellurate (ER-Te-57), selenite (AV-Te-18), selenate (ER-V-8), metavanadate (AV-V-4—brown, AV-V-5—black), and orthovanadate (ER-Te-41—brown, AV-V-19—grey/black) by isolates from deep sea hydrothermal vent worms. For Te oxide containing cultures, black coloration indicates reduction of oxide to elemental Te. Dissolved Se oxide color change from clear to red due to reduction to elemental Se. Change in color for V oxides is a result of reduction to lower oxidation state.

**Fig 2:**
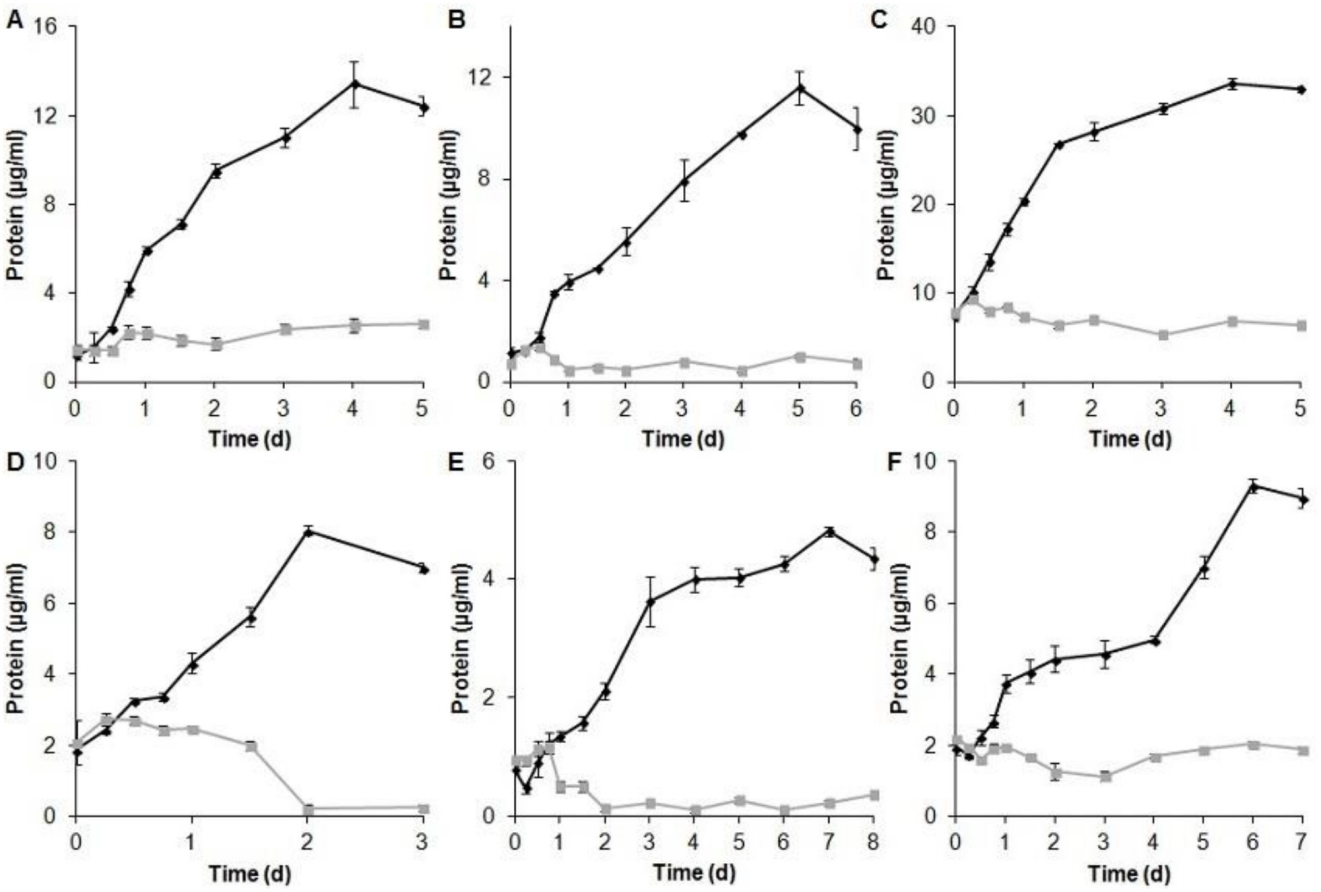
Growth as determined by protein production in the presence versus absence of metal(loid) oxides. A) Strain ER-Te-40B with tellurite; B) ER-Te-57 with tellurate; C) AV-Te-18 with selenite; D) ER-V-8 with selenate; E) AV-V-4 with metavanadate and F) ER-Te-41 with orthovanadate. ♦—With metal(loid) oxide; ▀—Without oxide. Error bars represent on standard deviation.

**Fig 3:**
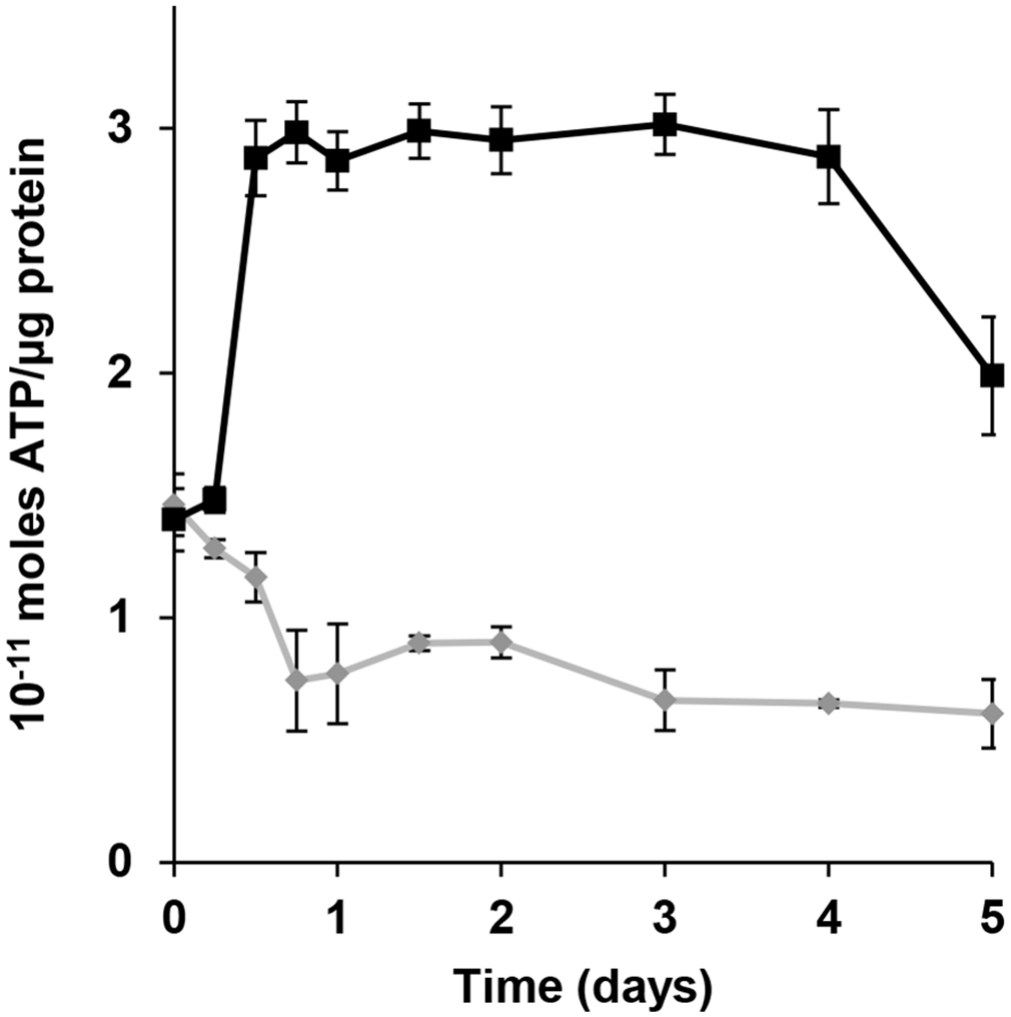
ATP production by cells of ER-Te-40B during anaerobic growth in presence of K_2_TeO_3_. A similar trend was seen for all remaining strains. ▀ —With metal(loid) oxide; ♦—Without oxide. Error bars represent on standard deviation.

Upon visual investigation for change of colorless water soluble oxides to colored elemental forms (black for Te, red for Se, and grey/black/brownish for V [4]) due to microbial activity, we found that under anaerobic conditions, all isolates but ER-V-1 were capable of reducing at least one (Fig 1), and many could use more than one of the oxides tested (Tables 1 and 2). While the color transformation obviously indicated the possibility of anaerobic respiration, experimental proof was required. For each oxide reduction group, one representative strain was chosen and protein levels with and without the oxide added to the growth medium were analyzed. Protein increased significantly in the presence of tellurite (5.6 fold), tellurate (10 fold), selenite (4.6 fold), selenate (4.3 fold), metavanadate (6.2 fold), and orthovanadate (4.8 fold), while no increase or decrease was observed in their absence (control tubes), due to no growth (Fig 2). There was no other electron acceptor in the medium, therefore, growth was clearly supported by metal(loid) oxides, confirming all strains were obtaining energy from anaerobic respiration. When ATP levels were monitored, they also increased during growth in the presence of the tested oxides, further confirming respiration (Fig 3). As discussed above, currently there are only 5, 17, and 6 strains confirmed to respire on Te, Se, and V oxides, respectively [4–19]. Our work adds significant numbers to the list (105, 85, 101, 1, 47, and 17 for tellurite, tellurate, selenite, selenate, metavanadate, and orthovanadate, respectively) and proves that anaerobic metal(loid) oxide based respiration is a quite common, well established mode of energy generation supporting life of symbiotic microorganisms associated with worms at deep sea hydrothermal vents. Interestingly, only one strain (ER-V-8) used selenate, which is possibly not a common form of Se in the habitat. Also, of the 107 isolates tested, 105 utilized more than one oxide. Hence, their metabolic capabilities are not narrowed on usage of a single element. Te oxides and selenite are preferred for respiration among those tested. Possibly these are the most prevalent oxides vent worms are exposed to, resulting in their widespread use. Another possibility might be that the pathway (enzyme(s)) expression needed for Te and Se oxide respiration is simpler than what is required for the V oxide based reactions. This idea is supported by the fact that Te and Se oxide reduction can be accomplished by the activity of a specific single enzyme [31–33], whereas V oxide based respiration involves multiple proteins [34]. As protein synthesis is energy intensive [35], for a cell to produce a single protein only in the presence of the inducing compound, instead of several, is less taxing.

**Table 1:** Range of metal(loid) oxides use for anaerobic respiration by strains symbiotically associated with tube worms at Axial Volcano.

	Metal(loid) Oxide							Metal(loid) Oxide					
Strain	TeO_3_^2-^	TeO_4_^2-^	SeO_3_^2-^	SeO_4_^2-^	VO_3_^-^	VO_4_^3-^	Strain	TeO_3_^2-^	TeO_4_^2-^	SeO_3_^2-^	SeO_4_^2-^	VO_3_^-^	VO_4_^3-^
AV-Se-12	+	+	+	-	-	-	AV-V-1	+	+	+	-	+	-
AV-Se-13	+	+	+	-	-	-	AV-V-10-1	+	+	+	-	-	-
AV-Se-15-dark	+	+	+	-	-	-	AV-V-10-2	+	+	+	-	+	-
AV-Se-16	+	+	+	-	-	-	AV-V-11	+	+	+	-	+	-
AV-Se-17	+	+	+	-	-	-	AV-V-12	+	+	+	-	+	-
AV-Se-18	+	+	+	-	-	-	AV-V-13	+	+	+	-	+	-
AV-Se-19	-	+	+	-	-	-	AV-V-14	+	+	+	-	+	+
AV-Se-2-dark	+	+	+	-	+	-	AV-V-15	+	+	+	-	+	+
AV-Se-3	+	+	+	-	-	-	AV-V-17	+	+	+	-	+	+
AV-Te-17	+	-	+	-	-	-	AV-V-19	+	+	+	-	+	+
AV-Te-18	+	+	+	-	+	+	AV-V-2	+	+	+	-	-	-
AV-Te-19	+	-	+	-	-	-	AV-V-20	+	-	-	-	-	-
AV-Te-20	+	-	+	-	-	-	AV-V-21	+	+	+	-	+	-
AV-Te-21-dark	+	+	+	-	-	-	AV-V-22	-	-	-	-	+	-
AV-Te-21-light	+	+	+	-	-	-	AV-V-23	+	-	+	-	+	-
AV-Te-22	+	-	+	-	-	-	AV-V-25	+	+	+	-	+	-
AV-Te-23-dark	+	+	+	-	-	-	AV-V-3	+	-	+	-	-	-
AV-Te-23-light	+	+	+	-	-	-	AV-V-4	+	+	+	-	+	-
AV-Te-24	+	+	+	-	+	+	AV-V-5	+	+	+	-	+	-
AV-Te-25	+	+	+	-	-	-	AV-V-6	+	+	+	-	-	-
AV-Te-26	+	+	+	-	-	-	AV-V-7	+	+	+	-	+	+
AV-Te-27	+	+	+	-	-	-							

+, Anaerobic respiration taking place;

-, No anaerobic respiration occurring.

**Table 2:** Range of metal(loid) oxides use for respiration by the Explorer Ridge vent worm symbionts.

	Metal(loid) Oxide							Metal(loid) Oxide					
Strain	TeO_3_^2-^	TeO_4_^2-^	SeO_3_^2-^	SeO_4_^2-^	VO_3_^-^	VO_4_^3-^	Strain	TeO_3_^2-^	TeO_4_^2-^	SeO_3_^2-^	SeO_4_^2-^	VO_3_^-^	VO_4_^3-^
ER-Se-1	+	+	+	-	-	-	ER-Te-51	+	+	+	-	-	-
ER-Se-13	+	-	+	-	-	-	ER-Te-52	+	+	+	-	-	+
ER-Se-14	+	+	+	-	-	-	ER-Te-53	+	+	+	-	-	-
ER-Se-15	+	-	+	-	+	-	ER-Te-54-dark	+	+	+	-	+	-
ER-Se-16	+	+	-	-	-	-	ER-Te-54-light	+	+	+	-	-	+
ER-Se-18	+	+	+	-	+	-	ER-Te-55	+	+	+	-	+	+
ER-Se-19-dark	+	+	-	-	-	-	ER-Te-56	+	+	+	-	+	-
ER-Se-2	+	-	+	-	-	-	ER-Te-57	+	+	+	-	+	+
ER-Se-20	+	+	+	-	-	-	ER-Te-58	+	+	+	-	+	-
ER-Se-21-dark	+	+	+	-	+	-	ER-Te-59	+	+	+	-	+	-
ER-Se-21-light	+	+	+	-	-	-	ER-Te-60	+	+	+	-	+	-
ER-Se-22-dark	+	-	+	-	-	-	ER-Te-61	+	+	+	-	-	-
ER-Se-22-light	+	-	+	-	-	-	ER-Te-63	+	-	+	-	-	-
ER-Se-3	+	-	+	-	-	-	ER-Te-64-fast	+	+	+	-	-	-
ER-Te-2-brown	+	-	+	-	-	-	ER-Te-64-slow	+	+	+	-	-	-
ER-Te-2-grey	+	-	+	-	-	-	ER-Te-65	+	+	+	-	-	-
ER-Te-40	+	+	+	-	+	-	ER-Te-66	+	+	+	-	-	-
ER-Te-40B	+	+	+	-	+	-	ER-V-10	+	+	+	-	+	-
ER-Te-41	+	+	+	-	-	+	ER-V-11	+	+	+	-	-	-
ER-Te-41B	+	+	+	-	+	-	ER-V-12	+	+	+	-	+	+
ER-Te-42	+	+	+	-	+	+	ER-V-13	+	+	+	-	-	-
ER-Te-42B-dark	+	+	+	-	-	-	ER-V-14	+	+	+	-	+	-
ER-Te-42B-light	+	+	+	-	-	+	ER-V-15	+	+	+	-	+	-
ER-Te-43	+	+	+	-	-	-	ER-V-2	+	-	-	-	+	-
ER-Te-44	+	+	+	-	+	-	ER-V-3	+	-	+	-	+	-
ER-Te-45	+	+	+	-	-	-	ER-V-4	+	+	-	-	+	-
ER-Te-46	+	+	+	-	+	-	ER-V-5	+	-	+	-	-	-
ER-Te-47	+	+	+	-	+	-	ER-V-6	+	+	+	-	+	+
ER-Te-48	+	+	+	-	+	+	ER-V-7	+	+	+	-	+	-
ER-Te-49	+	+	+	-	+	+	ER-V-8	+	+	+	+	+	-
ER-Te-50	+	+	+	-	-	-	ER-V-9	+	+	+	-	-	-
ER-Te-50-white	+	-	+	-	-	-	ER-V-1	-	-	-	-	-	-

+, Anaerobic respiration taking place;

-, No anaerobic respiration occurring.

### Phylogenetic analysis

Research into the microbial species makeup of specific locales in or around vents has been previously undertaken, focusing on low-temperature diffuse flow deep sea vents [36], vent plume waters [37], hydrothermal sediments [38], and microbial mats covering vent chimneys [39–41]. These studies have shown extremely diverse bacterial populations that can differ significantly between neighbouring vents. The metabolic diversity has also received some attention [42] as well as the epibionts of vent inhabitants such as sulfide tube and tube worms [23, 43]. *Riftia pachyptila* possesses chemolithoautotrophic, sulphur-oxidizing endosymbionts, which autotrophically fix carbon dioxide, using reduced sulphur compounds from vent fluids as electron donors, thereby cleaning the blood from toxic sulfide, and synthesizing organic compounds for their host [22]. However, the presence, and especially diversity, of metal(loid) oxide respiring bacteria, which can obviously help to remove toxic metal(loid)s dissolved in surrounding water and, therefore, detoxify the blood of their hosts, has not been considered yet. Our collection of strains is shedding some light on this important component of symbiotic populations.

**Fig 4:**
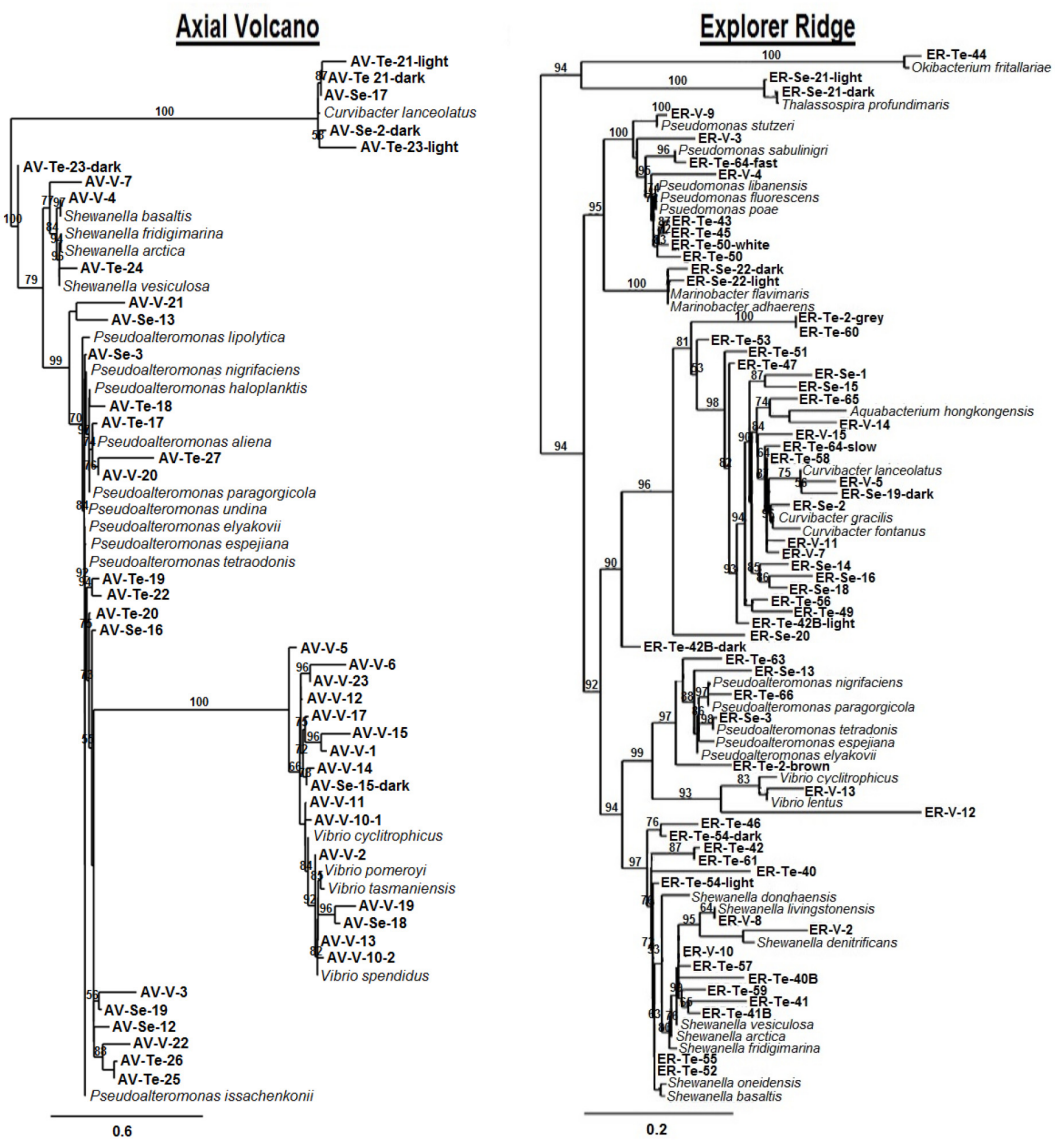
Maximum likelihood phylogenetic tree of strains based on partial 16S rRNA gene sequences (avg. 400 bp) showing position of new metal(loid) respiring isolates from Axial Volcano (Bar represents 0.6%) and Explorer Ridge (Bar represents 0.2%) tube worms and their closest neighbors.

**Fig 5:**
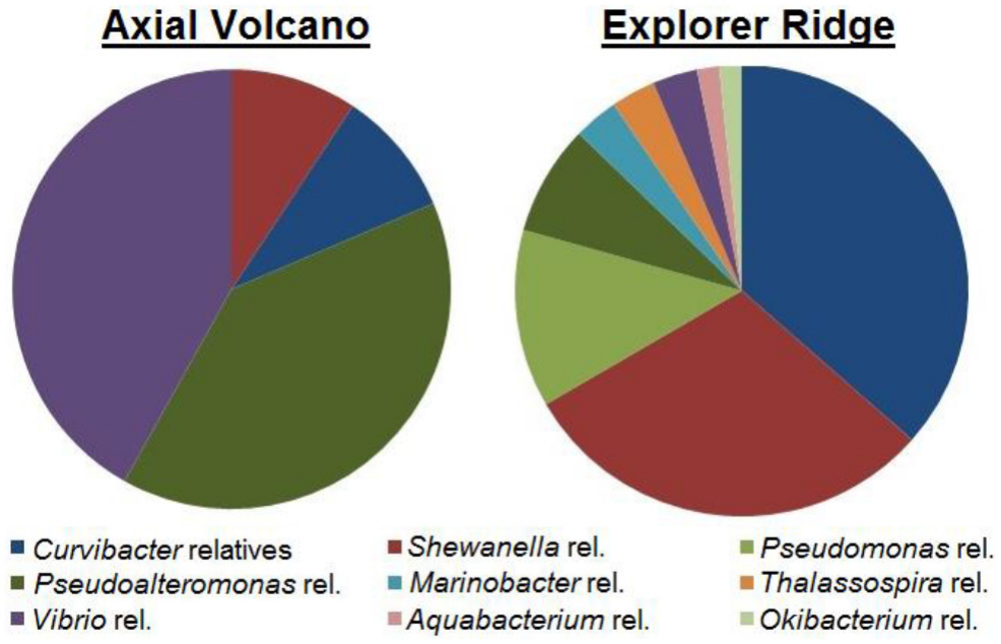
Distribution of metal(loid) oxide respiring epibionts of tube worms at Axial Volcano and Explorer Ridge.

Three strains (ER-Te-48, ER-V-6, and AV-V-25) had been previously sequenced [4]. Partial 16S rRNA gene sequencing of the 103 remaining isolates revealed a highly diverse group (Fig 4). Each sample taken from different animals had a distinct phylogenetic suite of microbes (Fig 5). Epibionts originating from the vent worms living at Axial Volcano were dominated by *Vibrio* (41.9%) and *Pseudoalteromonas* (39.5%) relatives, with *Curvibacter* (9.3%) and *Shewanella* (9.3%) relatives making up the remainder. The Explorer Ridge tube worm samples had different distribution and composition. The isolates had greater variety, dominated by *Curvibacter* (36.5%) and *Shewanella* (30.2%) relatives. The remaining organisms were comprised of *Pseudomonas* (12.7%), *Pseudoalteromonas* (7.9%), *Marinobacter* (3.2%), *Thalassospira* (3.2%), *Vibrio* (3.2%), *Aquabacterium* (1.6%), and *Okibacterium* (1.6%) relatives. Some of these genera are already known for their metal(loid) oxide respiring capabilities [4–19]. *Shewanella* species are metabolically diverse and versatile in regards to metal resistance and respiration [34], the best characterized being *S*. *oneidensis*, MR-1. Therefore, it is not surprising that they comprise a significant proportion of the isolates (39.5%). The same can be said about the *Pseudomonas* relatives. While they were not as abundant (12.7% of total isolates in samples), the genus is known to respire on selenate (*Pseudomonas stutzeri*, pn1) [9] and metavanadate (*P*. *isachenkovii* and *P*. *vanadiumreductans*) [17]. Our work indicates both genera contain many members capable of metal(loid) oxide respiration. In concurrence with previous publication [36], our data demonstrate that neighbouring deep sea habitats, including symbiotic bacterial populations of worms, may vary significantly in terms of composition. Comparing the two samples in our hands, we see the majority of isolates are ɤ-proteobacteria, however, the dominant genera associated with Axial Volcano tube worms comprise a minor fraction in the Explorer Ridge hosts. Such unequal distribution is likely due to the nature of this extraordinary environment. Deep ocean vent habitats are unstable, with features such as temperature, flow rate, water composition, and overall activity being highly variable and very often changing [44]. Radical fluctuations in parameters as a result of hydrothermal activity can lead to abolishment of life in the vent vicinity [45]. However, following these drastic changes, life is quick to recover. Lower bacterial diversity could be a result of a recent change in vent activity, causing re-establishment of life in the surrounding area. The subsequent primary microbial succession would lead to a more narrow range of microorganisms. Faster growing bacteria, such as *Vibrio* [46], would dominate during initial colonization, followed by a progression of slower growing microbes, ultimately resulting in a climax population with greater variety. The more mature diverse population was seen in worms at ER, which had an increased number of genera represented (9 total), while the population of AV worms was comprised of only 4, the majority of which are *Vibrio* relatives. A second possibility may be related to the age of the worms. Similar to above, if a worm is young, it will not have a mature climax population of epibionts. However, an older worm is more likely to have a much greater diversity of bacteria residing in/on it. Therefore, the age of the worm sampled at each location may be the cause of the difference observed between ER and AV symbionts, not necessarily a drastic geological event, causing a major disturbance to the ecosystem.

The diversity of symbionts in our study is similar to those found in other worms living in related habitats. Bacterial populations associated with *Lamellibrachia* sp. and *Escarpia southwardae*, from cold seeps in the eastern Mediterranean, are also dominated by ɤ-proteobacteria [47, 48]. However, despite this similarity in sharing this ɤ subclass affiliation, the genera and species composition does vary. When looking at the microbial populations of the tubes, there is little similarity to our results obtained from tissue samples. The biofilms on tubes of *R*. *pachyptila* are comprised of primarily ε-proteobacteria, as are those of *P*. *sulfincola* [49, 50]. It is interesting that *P*. *sulfincola* tissue has a much different group of epibiotic bacteria in comparison to its tube. Obviously microbes inhabiting tubes do not necessarily colonize the body of the worm, creating significantly different communities even in such close proximity.

Lastly, the greatly varying sequence similarities to known species (from as low as 90.6 to as high as 100%) indicate diverse microbial populations (Table 3). Surprisingly, two strains (ER-Se-20 and ER-Te-44) are close relatives of non-marine bacteria (*Aquabacterium* and *Okibacterium*, respectively). The first genus is freshwater [51] and the latter is comprised of a sole aerobic species associated with plant seeds [52]. Also, 28 *Curvibacter* relatives were identified (Table 3), even though all published members have been isolated from freshwater wells [53, 54]. Clearly, sequencing hints on several potentially new taxonomic genera and species inviting further study for definitive identification.

**Table 3:** Nearest phylogenetic relative for each isolate as determined by partial 16S rRNA gene sequencing.

Strain	Nearest Relative (% 16S rRNA Similarity)	Strain	Nearest Relative (% 16S rRNA Similarity)
AV-Se-12	*Pseudoalteromonas espejiana* (96.2)	ER-Se-21-light	*Thalassospira profundimaris* (99.7)
AV-Se-13	*Pseudoalteromonas* issachenkonii (91.3)	ER-Se-22-dark	*Marinobacter adhaerens* (98.2)
AV-Se-15 dark	*Vibrio splendidus* (98.3)	ER-Se-22-light	*Marinobacter flavimaris* (98.6)
AV-Se-16	*Pseudoalteromonas elyakovii* (99.1)	ER-Se-3	*Pseudoalteromonas nigrifaciens* (98.5)
AV-Se-17	*Curvibacter lanceolatus* (98.3)	ER-Te-2-brown	*Pseudoalteromonas paragorgicola* (93.5)
AV-Se-18	*Vibrio cyclitrophicus* (97.1)	ER-Te-2-grey	*Curvibacter gracilis* (95.9)
AV-Se-19	*Pseudoalteromonas elyakovii* (97.3)	ER-Te-40	*Shewanella frigidimarina* (92.5)
AV-Se-2-dark	*Curvibacter lanceolatus* (97.9)	ER-Te-40B	*Shewanella vesiculosa* (92.4)
AV-Se-3	*Pseudoalteromonas paragorgicola* (99.5)	ER-Te-41	*Shewanella vesiculosa* (95.6)
AV-Te-17	*Pseudoalteromonas elyakovii* (98.0)	ER-Te-41B	*Shewanella vesiculosa* (98.5)
AV-Te-18	*Pseudoalteromonas nigrifaciens* (96.3)	ER-Te-42	*Shewanella arctica* (94.2)
AV-Te-19	*Pseudoalteromonas haloplanktis* (98.0)	ER-Te-42B-dark	*Shewanella oneidensis* (93.4)
AV-Te-20	*Pseudoalteromonas tetraodonis* (99.0)	ER-Te-42B-light	*Curvibacter lanceolatus* (95.8)
AV-Te-21-dark	*Curvibacter lanceolatus* (98.6)	ER-Te-43	*Pseudomonas libanensis* (98.3)
AV-Te-21-light	*Curvibacter lanceolatus* (96.8)	ER-Te-44	*Okibacterium fritillariae* (98.2)
AV-Te-22	*Pseudoalteromonas undina* (96.8)	ER-Te-45	*Pseudomonas poae* (98.6)
AV-Te-23-dark	*Shewanella arctica* (95.5)	ER-Te-46	*Shewanella oneidensis* (96.3)
AV-Te-23-light	*Curvibacter lanceolatus* (92.1)	ER-Te-47	*Curvibacter lanceolatus* (96.1)
AV-Te-24	*Shewanella vesiculosa* (95.2)	ER-Te-48	*Shewanella frigidimarina*^a^
AV-Te-25	*Pseudoalteromonas haloplanktis* (93.4)	ER-Te-49	*Curvibacter lanceolatus* (94.6)
AV-Te-26	*Pseudoalteromonas espejiana* (94.4)	ER-Te-50	*Pseudomonas fluorescens* (96.5)
AV-Te-27	*Pseudoalteromonas tetraodonis* (90.6)	ER-Te-50-white	*Pseudomonas libanensis* (98.5)
AV-V-1	*Vibrio tasmaniensis* (95.1)	ER-Te-51	*Curvibacter gracilis* (93.9)
AV-V-10-1	*Vibrio splendidus* (98.4)	ER-Te-52	*Shewanella basaltis* (99.7)
AV-V-10-2	*Vibrio cyclitrophicus* (98.7)	ER-Te-53	*Curvibacter lanceolatus* (92.8)
AV-V-11	*Vibrio cyclitrophicus* (99.6)	ER-Te-54-dark	*Shewanella basaltis* (97.7)
AV-V-12	*Vibrio splendidus* (99.1)	ER-Te-54-light	*Shewanella basaltis* (99.0)
AV-V-13	*Vibrio splendidus* (99.9)	ER-Te-55	*Shewanella oneidensis* (99.0)
AV-V-14	*Vibrio splendidus* (96.7)	ER-Te-56	*Curvibacter lanceolatus* (97.3)
AV-V-15	*Vibrio pomeroyi* (91.4)	ER-Te-57	*Shewanella vesiculosa* (98.3)
AV-V-17	*Vibrio cyclitrophicus* (98.6)	ER-Te-58	*Curvibacter lanceolatus* (100)
AV-V-19	*Vibrio cyclitrophicus* (94.3)	ER-Te-59	*Shewanella vesiculosa* (97.4)
AV-V-2	*Vibrio splendidus* (100)	ER-Te-60	*Curvibacter fontanus* (95.4)
AV-V-20	*Pseudoalteromonas aliena* (98.1)	ER-Te-61	*Shewanella donghaensis* (97.3)
AV-V-21	*Pseudoalteromonas elyakovii* (91.7)	ER-Te-63	*Pseudoalteromonas elyakovii* (96.3)
AV-V-22	*Pseudoalteromonas haloplanktis* (91.3)	ER-Te-64-fast	*Pseudomonas sabulinigri* (98.5)
AV-V-23	*Vibrio splendidus* (98.6)	ER-Te-64-slow	*Curvibacter lanceolatus* (98.2)
AV-V-25	*Vibrio pomeroyi*^a^	ER-Te-65	*Curvibacter lanceolatus* (95.4)
AV-V-3	*Pseudoalteromonas lipolytica* (90.8)	ER-Te-66	*Pseudoalteromonas tetradonis* (99.6)
AV-V-4	*Shewanella basaltis* (99.7)	ER-V-10	*Shewanella vesiculosa* (99.7)
AV-V-5	*Vibrio tasmaniensis* (96.7)	ER-V-11	*Curvibacter lanceolatus* (97.5)
AV-V-6	*Vibrio cyclitrophicus* (92.6)	ER-V-12	*Vibrio cyclitrophicus* (92.7)
AV-V-7	*Shewanella frigidimarina* (94.7)	ER-V-13	*Vibrio lentus* (95.8)
ER-Se-1	*Curvibacter lanceolatus* (94.2)	ER-V-14	*Curvibacter lanceolatus* (95.6)
ER-Se-13	*Pseudoalteromonas espejiana* (95.4)	ER-V-15	*Curvibacter lanceolatus* (95.3)
ER-Se-14	*Curvibacter lanceolatus* (95.9)	ER-V-2	*Shewanella denitrificans* (97.5)
ER-Se-15	*Curvibacter lanceolatus* (94.6)	ER-V-3	*Pseudomonas marincola* (97.3)
ER-Se-16	*Curvibacter lanceolatus* (94.5)	ER-V-4	*Pseudomonas fluorescens* (95)
ER-Se-18	*Curvibacter lanceolatus* (96.7)	ER-V-5	*Curvibacter lanceolatus* (97.0)
ER-Se-19-dark	*Curvibacter lanceolatus* (96.4)	ER-V-6	*Shewanella frigidimarina*^a^
ER-Se-2	*Curvibacter lanceolatus* (97.8)	ER-V-7	*Curvibacter lanceolatus* (97.9)
ER-Se-20	*Aquabacterium hongkongensis* (92.9)	ER-V-8	*Shewanella livingstonensis* (100)
ER-Se-21-dark	*Thalassospira profundimaris* (99.9)	ER-V-9	*Pseudomonas stutzeri* (99.7)

^a^Csotonyi et al 2006.

In summary, it has been a long held belief that Te is a biologically insignificant element and its oxides were only considered as strong toxins to life. However, the recent discovery of bacteria capable of incorporating its oxides into metabolic pathways indicates otherwise [4]. The discoveries of our previous work and this study show that Te in some habitats definitely supports life. Around black smokers in particular, metal(loid) oxide respiration is not simply an ability possessed by a few select bacteria, but is an established method of energy generation for a vast diversity of microbes. Very importantly, such symbiotic microbial suites provide protection from toxic metal(loid) compounds present in vent fluids and, therefore, diffused into the host blood, by their removal via anaerobic respiration. It has long been known that these worms have an important symbiotic relationship with sulfur bacteria [22], which remove toxic sulfides from the blood by its conversion. Our data suggests a similar positive relationship may exist between worms and metal(loid) oxide converting bacteria described in this paper.

## Supporting Information

S1 Table Genbank accession numbers for 16S rRNA gene sequences reported in this study. (DOCX)
