# Supplementary material for: A Diverse Community of Metal(loid) Oxide Respiring Bacteria Is Associated with Tube Worms in the Vicinity of the Juan de Fuca Ridge Black Smoker Field
Source: PLoS One. 2016 Feb 25;11(2):e0149812. doi: 10.1371/journal.pone.0149812 (PMC4767881; doi:10.1371/journal.pone.0149812)
Supplement: S1 Table — (DOCX) [file pone.0149812.s001.docx]

**Table S1.** Genbank accession numbers for 16S rRNA gene sequences obtained in this study

| **Strain** | **Sequence Accession Number** | **Strain** | **Sequence Accession Number** |
| --- | --- | --- | --- |
| AV-Se-12 | KT277103 | ER-Se-21-light | KT277155 |
| AV-Se-13 | KT277104 | ER-Se-22-dark | KT277156 |
| AV-Se-15-dark | KT277105 | ER-Se-22-light | KT277157 |
| AV-Se-16 | KT277106 | ER-Se-3 | KT277158 |
| AV-Se-17 | KT277107 | ER-Te-2-brown | KT277159 |
| AV-Se-18 | KT277108 | ER-Te-2-grey | KT277160 |
| AV-Se-19 | KT277109 | ER-Te-40 | KT277161 |
| AV-Se-2-dark | KT277110 | ER-Te-40B | KT277162 |
| AV-Se-3 | KT277111 | ER-Te-41 | KT277163 |
| AV-Te-17 | KT277112 | ER-Te-41B | KT277164 |
| AV-Te-18 | KT277113 | ER-Te-42 | KT277165 |
| AV-Te-19 | KT277114 | ER-Te-42B-dark | KT277166 |
| AV-Te-20 | KT277115 | ER-Te-42B-light | KT277167 |
| AV-Te-21-dark | KT277116 | ER-Te-43 | KT277168 |
| AV-Te-21-light | KT277117 | ER-Te-44 | KT277169 |
| AV-Te-22 | KT277118 | ER-Te-45 | KT277170 |
| AV-Te-23-dark | KT277119 | ER-Te-46 | KT277171 |
| AV-Te-23-light | KT277120 | ER-Te-47 | KT277172 |
| AV-Te-24 | KT277121 | ER-Te-49 | KT277173 |
| AV-Te-25 | KT277122 | ER-Te-50 | KT277174 |
| AV-Te-26 | KT277123 | ER-Te-50-white | KT277175 |
| AV-Te-27 | KT277124 | ER-Te-51 | KT277176 |
| AV-V-1 | KT277125 | ER-Te-52 | KT277177 |
| AV-V-10-1 | KT277126 | ER-Te-53 | KT277178 |
| AV-V-10-2 | KT277127 | ER-Te-54-dark | KT277179 |
| AV-V-11 | KT277128 | ER-Te-54-light | KT277180 |
| AV-V-12 | KT277129 | ER-Te-55 | KT277181 |
| AV-V-13 | KT277130 | ER-Te-56 | KT277182 |
| AV-V-14 | KT277131 | ER-Te-57 | KT277183 |
| AV-V-15 | KT277132 | ER-Te-58 | KT277184 |
| AV-V-17 | KT277133 | ER-Te-59 | KT277185 |
| AV-V-19 | KT277134 | ER-Te-60 | KT277186 |
| AV-V-2 | KT277135 | ER-Te-61 | KT277187 |
| AV-V-20 | KT277136 | ER-Te-63 | KT277188 |
| AV-V-21 | KT277137 | ER-Te-64-fast | KT277189 |
| AV-V-22 | KT277138 | ER-Te-64-slow | KT277190 |
| AV-V-23 | KT277139 | ER-Te-65 | KT277191 |
| AV-V-3 | KT277140 | ER-Te-66 | KT277192 |
| AV-V-4 | KT277141 | ER-V-10 | KT277193 |
| AV-V-5 | KT277142 | ER-V-11 | KT277194 |
| AV-V-6 | KT277143 | ER-V-12 | KT277195 |
| AV-V-7 | KT277144 | ER-V-13 | KT277196 |
| ER-Se-1 | KT277145 | ER-V-14 | KT277197 |
| ER-Se-13 | KT277146 | ER-V-15 | KT277198 |
| ER-Se-14 | KT277147 | ER-V-2 | KT277199 |
| ER-Se-15 | KT277148 | ER-V-3 | KT277200 |
| ER-Se-16 | KT277149 | ER-V-4 | KT277201 |
| ER-Se-18 | KT277150 | ER-V-5 | KT277202 |
| ER-Se-19-dark | KT277151 | ER-V-7 | KT277203 |
| ER-Se-2 | KT277152 | ER-V-8 | KT277204 |
| ER-Se-20 | KT277153 | ER-V-9 | KT277205 |
| ER-Se-21-dark | KT277154 |  |  |
